# Move on up: Fingertip forces and felt heaviness are modulated by the goal of the lift

**DOI:** 10.3758/s13414-019-01703-w

**Published:** 2019-04-04

**Authors:** Gavin Buckingham, Heather Donald

**Affiliations:** 1 Sport and Health Sciences, University of Exeter, Exeter, EX2 4LU UK; 2 Department of Psychology, Heriot-Watt University, Edinburgh, UK

**Keywords:** Perception and Action, Haptics

## Abstract

When we interact with objects, we usually do so for a purpose. It is well known that the specific goal of an action can have a substantial effect on initial reach kinematics. No research, however, has examined the effect that the goal of a lift can have on the fingertip forces and perception of object weight when picking up an object to move it. Here, we report a study in which participants were asked to move objects laterally to a higher platform, to a lower platform, or to a platform of the same height. The objects were rated, on average, as feeling heavier after they were moved to a higher platform than after they were moved to a lower platform or to a platform of the same height. Furthermore, participants gripped and lifted with more force, and used higher rates of force, when moving objects to a higher platform compared with moving it to a platform of the same height. These findings suggest that the goal of movement in the context of object interaction may affect how heavy an object feels and the way in which it is lifted.

We interact with objects hundreds of times every day. When lifting objects, even ones we have not touched before, we typically apply forces in a predictive manner, with fingertip forces and pre-lift-off force rates reflecting the object’s expected weight (Flanagan & Johansson, 2011). Although we typically grasp objects to use them in some way, many of our interactions with objects are perceptual in nature, with the goal of evaluating nonvisual properties such as weight. Interestingly, many factors can influence how heavy an object feels when it is lifted. For example, it is well known that an object will feel heavier when it is cold than when it is at room temperature (Ross & Murray, 1978). Furthermore, the surface friction of an object can influence how heavy it feels, such that slippery objects feel heavier than nonslippery objects do (Flanagan, Wing, Allison, & Spenceley, 1995)—an effect caused by the increased grip force required to maintain an appropriate friction coefficient when lifting slippery objects. In fact, how an object is gripped and lifted can influence how heavy it feels. For example, it is easily demonstrated that lifting an object rapidly will make it feel less heavy than it would feel when it is lifted slowly. Similarly, lifting an object with a wider grip aperture, or using more digits to grip the object surface, can make it feel heavier than it actually is (Flanagan & Bandomir, 2000).

In addition to these low-level factors, a range of higher level influences on the perception of object weight results in dramatic weight illusions. The most famous example of how humans misperceive object weight can be experienced with the size–weight illusion (SWI), in which small objects feel substantially heavier than identically weighted large objects (Charpentier, 1891). This illusory weight difference has been shown to be unrelated to sensorimotor factors (Flanagan & Beltzner, 2000; Grandy & Westwood, 2006; Mon-Williams & Murray, 2000) and is instead thought to reflect the role of cognitive expectations on our perception of heaviness. A lifetime of experiencing the positive correlation between size and mass cause lifters to expect the large object to outweigh the small object, and therefore to experience it as lighter than expected and vice versa (for review, see Buckingham, 2014). Indeed, even a single object can be made to feel it is of a substantially different weight if an individual merely expects to be lifting something heavier or lighter than the object they eventually interact with (Buckingham & Goodale, 2010; Buckingham, Ranger, & Goodale, 2011). This effect is ubiquitous, having been demonstrated in a wide range of populations—from children as young as 2 years (Robinson, 1964), to patients with unilateral brain injury (Buckingham, Bieńkiewicz, Rohrbach, & Hermsdörfer, 2015a), to blind human echolocators (Buckingham, Milne, Byrne, & Goodale, 2015b)—and can only be influenced by thousands of trials of perceptual learning (Flanagan, Bittner, & Johansson, 2008). Similar, albeit much smaller, effects can be experienced with the material–weight illusion, in which objects that appear to be made from a light material feel slightly heavier than objects that appear to be made from a more dense material (Buckingham, Cant, & Goodale, 2009; Buckingham et al., 2011; Ellis & Lederman, 1999). These weight illusions are considered to be a unique instance of an individual’s perception reflecting a combination of sensory input with the inverse of perceptual prior expectations (Brayanov & Smith, 2010).

The nature of the prior expectation that drives these weight illusions is, however, far from clear. One reason for this lack of a mechanistic understanding might stem from the tasks typically employed in perceptual weight-judgement tasks. In the majority of weight perception studies, the participant is simply told to lift and report (or compare with a standard) the weight of an object, before replacing it on the table surface. In our daily lives, however, actions typically have a goal with an end state that is distinct from the originating movement. Indeed, very few studies have examined weight perception in the context of a more natural, goal-directed movement. Interestingly, a growing body of work in the context of reach-to-grasp movements suggests that the end-state goal of an action can affect how the movement itself is planned. Typically, these studies assess movement kinematics during early phases of a grasping movement, showing that the likely end posture of the grasp will affect the start posture of the grasp, with individuals typically prioritising end-state comfort over initial-state comfort (for review, see Rosenbaum, Chapman, Weigelt, Weiss, & van der Wel, 2012). It remains unclear, however, the extent to which premovement parameters, such fingertip-force parameterization, and subsequent experiences of heaviness (Flanagan et al., 1995) can be affected by movement goals. Understanding the relationship between the prior expectations that can drive heaviness perceptions and the goal-directed effects that can influence grasp planning might shed light on the interplay between hedonic perception and motor planning.

In order to better understand the nature of the expectations that appear to influence heaviness perception, we examined how weight perception is affected by varying the goal of lift. To this end, we developed a goal-directed manipulation related to the expected effort requirements of the movement in each condition. In order to manipulate the apparent effort requirements of the lift, without altering other perceptual properties of the stimuli, we instructed participants to lift and transport hand-held objects laterally to a platform that varied in height from trial to trial. In three randomised conditions, the objects were either moved to a position of the same height as their starting position, to a higher platform, or to a lower platform. We reasoned that participants would expect that moving an object to a higher platform would be more difficult (i.e., require more effort) than moving the object laterally or to a lower platform. If these goal-related expectations influenced perceptions of heaviness in a way analogous to object size or apparent material properties, it seems likely that an object being moved to a higher shelf might feel less heavy than it would if it was moved to a lower shelf (i.e., because the lifter expects more work to be done). To determine how, if at all, the perceptual consequences of the goal of the lift interact with the expectation effects seen in the classic weight-illusion paradigms, participants moved objects that induced the SWI (varying sizes, with identical mass). We also measured fingertip forces and force rates to evaluate whether any such perceptual effects might be driven by the effects of movement goals on sensorimotor prediction during lifting.

## Materials and method

Thirty-two right-handed university students (13 male, age range: 18–33 years) were recruited for this study through an online recruitment system in return for course credit. Participants all gave written consent before taking part in this study, and all procedures were approved by the Heriot-Watt University Ethics Committee.

**Fig. 1:**
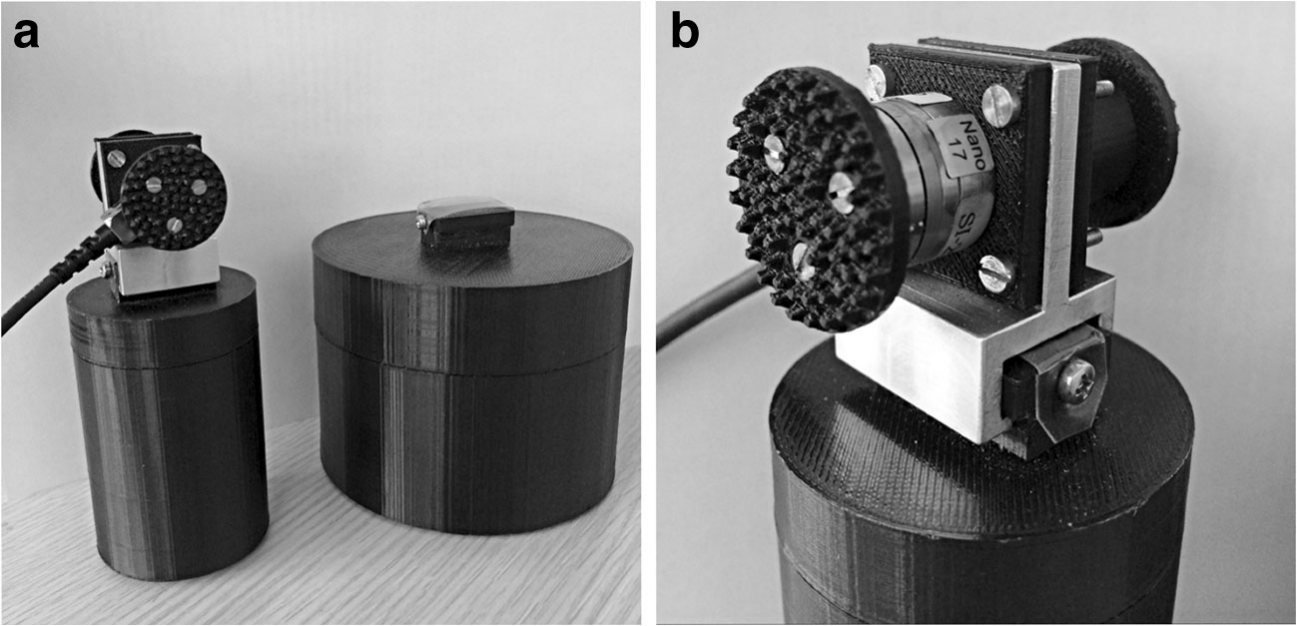
The small and large cylinders lifted by participants during the experimental trials (**a**), and a close-up of the handle used to lift the object with the thumb and index finger on the grasp pads (**b**)

Participants performed a simple object-lifting task requiring them to lift an object from a slightly raised platform and move it laterally to a different platform which was higher, lower, or the same height as the starting platform in a blocked, repeated-measures design. In the experiment, participants lifted a pair of cylinders with the same height (7.5 cm) and mass (400 g), as one another, but different diameters (small: 5 cm and large: 10 cm; see Fig. 1a). When these stimuli are lifted, individuals typically experience the large object as feeling less heavy than the smaller object (i.e., the size–weight illusion). Participants grasped and lifted each object via a custom-built aluminium and plastic handle containing a Nano17 six-axis force-torque sensor, which could be easily attached to each object through a central mount (see Fig. 1b).

**Fig. 2:**
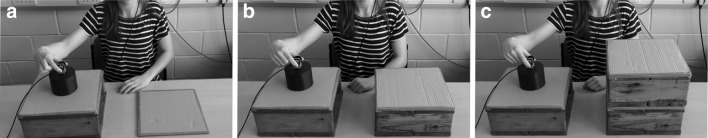
The configuration for the three experimental movement goal conditions. **a** The lower movement. **b** The same-height movement. **c** The higher movement

Combinations of equally sized stackable wooden boxes (W × H × D: 27.5 cm × 13.5 cm × 27.5 cm), and a cardboard square with the same surface area as these boxes, were used to create the starting platform (one box high) on the right side of the participant’s body midline, and the three varying-height goal platforms 13.5 cm to the left of starting platform. The lower ‘platform’ (see Fig. 2a) was marked with a cardboard square on the table surface, requiring participants to make a lateral movement that ended 13.5 cm lower than the start. The same-height platform was, like the starting platform, one box high (see Fig. 2b), thus requiring a movement that started and ended from the same height. The higher platform (see Fig. 2c) was two boxes high (i.e., twice as high as the starting platform), requiring a lateral movement that ended 13.5 cm higher than the start position. Identical cardboard squares were also used to cover each of wooden platforms to maintain uniformity in start and end surfaces.

Each participant began the study with a practice lift using a nonexperimental object (a wooden cube) to ensure they used the correct lifting technique, followed by 60 lifts—10 lifts per object per condition in one of four pseudorandomised orders generated with Microsoft Excel, two of which were reversed with respect to object size compared with the other pair. PLATO shutter goggles (Translucent Technologies, Toronto, Canada) were used to obscure the participants’ vision between lifts. Upon the PLATO shutter goggles opening, alongside an auditory cue being played, participants reached out and moved the object from the starting platform laterally from right to left onto a higher platform, to a platform of the same height, or to a lower platform (the cardboard square on the surface of the table). All participants performed the lifts using their thumb and index finger on their right hand in a smooth and controlled motion, as instructed in the practice trials. Once they had released the cylinder, participants gave an unconstrained perceptual rating of how heavy the object they just lifted felt to them by giving a numerical value (i.e., an absolute magnitude estimation; Zwislocki & Goodman, 1980).

The data from the force transducers were processed using a custom-written MATLAB script (https://sites.google.com/site/obintlab/wiki/data-processing). The force orthogonal to the grasp pad surface was defined as grip force, and the vertical and lateral forces (i.e., parallel to the surface of the grasp pad) were vector summed and defined as load force. These force traces were smoothed with a 14-Hz low-pass Butterworth filter and differentiated with a 5-point central difference equation to yield grip-force rate and load-force rate. The peak values of grip force (pGF), load force (pLF), grip force rate (pGFR) and load force rate (pLFR) were used as our dependant variable related to sensorimotor prediction. Traces for each of these measures were screened individually to ensure that the correct peak was chosen, averaged across the 10 lifts of each size/movement goal combination, and examined with a 2 (object size) × 3 (movement goal) repeated-measures ANOVA. Violations of sphericity were addressed with the Greenhouse–Geisser correction. Significant main effects and interactions were followed by Bonferroni-corrected post hoc comparisons. The perceptual ratings of heaviness were transformed to *Z* scores at an individual level, and analysed in the same way as the fingertip-force data. All statistical analyses were performed in JASP 0.9. The raw data, as well as the lifting orders, can be found here: https://osf.io/pmq52/.

## Results

### Perception of object heaviness

**Fig. 3:**
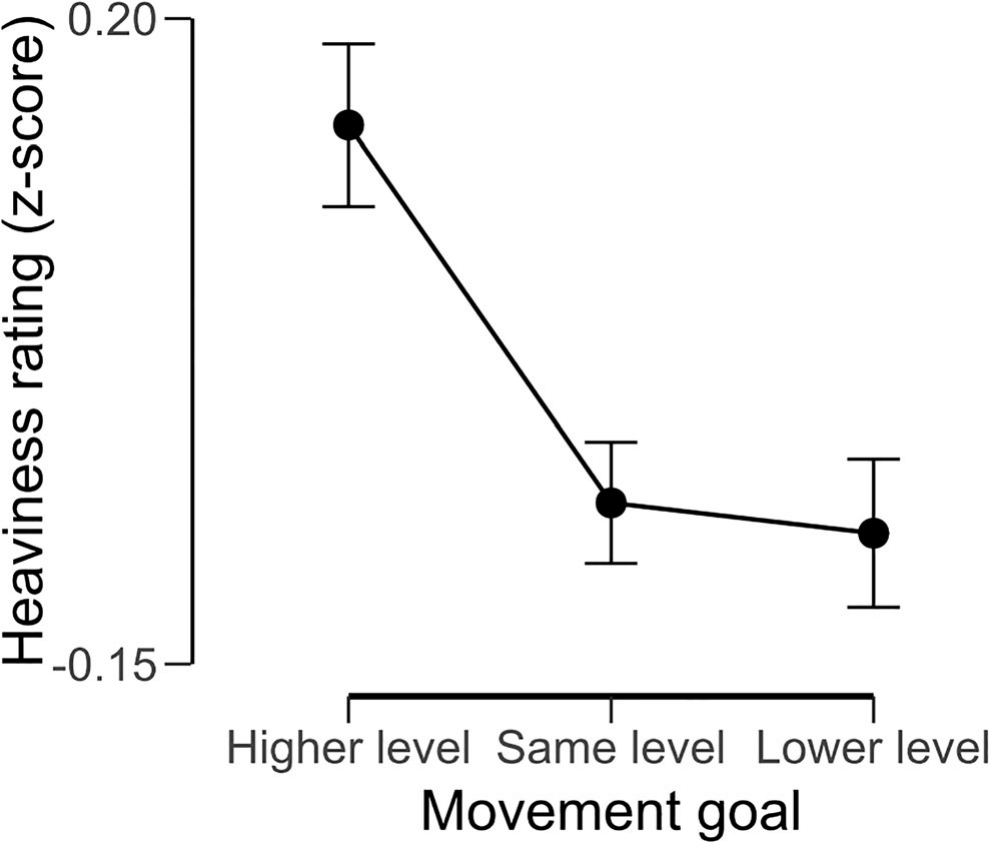
The heaviness ratings normalized to a *z* distribution given after moving the objects onto platforms of different heights, averaged across the small and large objects. Error bars show standard error of the mean, pooled across repeated-measures factors

With the perceptual ratings of heaviness, we observed a main effect of object size, *F*(1, 31) = 590.64, *p* < .001, ω^2^ = 0.95, suggesting that participants experienced a robust size–weight illusion (small objects felt an average of 1.49 units heavier than large objects). We also observed a main effect of movement goal, *F*(2, 62) = 9.82, *p* < .001, ω^2^ = 0.22. Post hoc comparisons (see Fig. 3) highlighted that moving the object to a higher platform made the objects feel heavier than if they were moved to a lower platform (*p* = .004, Cohen’s *d* = 0.62) or a platform of the same height (*p* = .002, Cohen’s *d* = 0.67). There was no difference in perceived heaviness when the object was moved to a lower platform compared with one of the same height (*p* > .99, Cohen’s *d* = 0.06). No interaction was observed between object size and movement goal, *F*(2, 62) = 0.07, *p* = .93, ω^2^ < .001.

### Peak forces used to lift objects

**Fig. 4:**
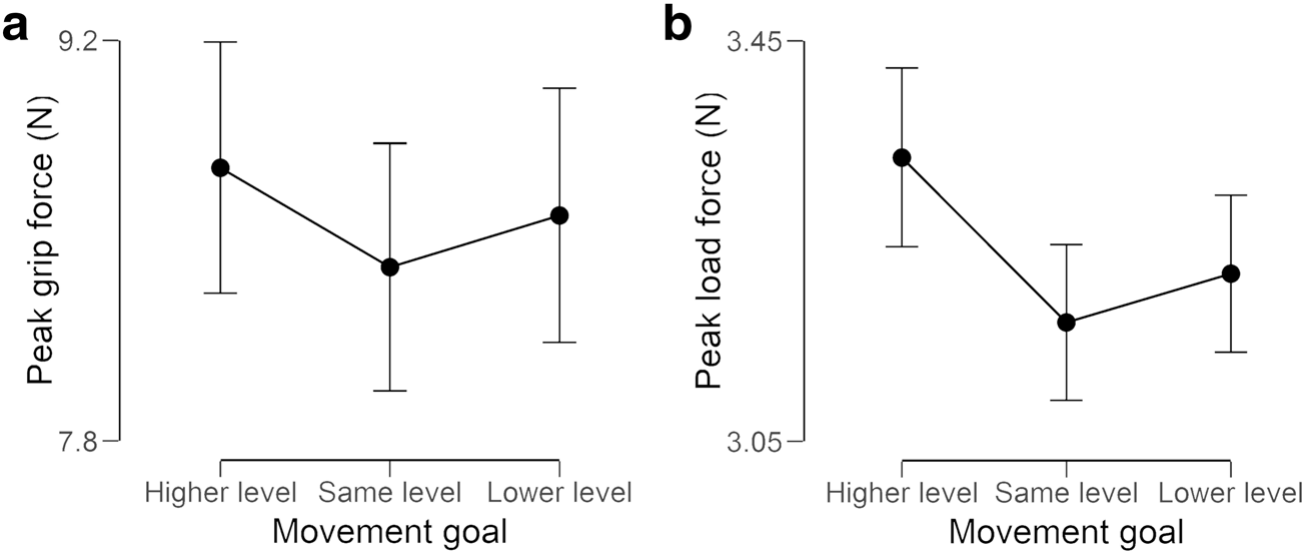
The (**a**) peak grip forces and (**b**) peak load forces used to move objects towards platforms of different heights, averaged across the small and large objects. Error bars show standard error of the mean, pooled across repeated-measures factors

With the pGF data, we observed a main effect of object size, *F*(1, 31) = 15.63, *p* < .001, ω^2^ = 0.016, suggesting that participants gripped the large objects with more force than they did the small objects (average mean difference of 0.52 N). We also observed a main effect of movement goal, *F*(2, 62) = 5.66, *p* = .006, ω^2^ = 0.004. Post hoc comparisons (see Fig. 4a) showed that participants gripped harder when moving the object to a higher platform than to a platform of the same height (*p* < .001, Cohen’s *d* = 0.76), but not to a lower platform (*p* = .47). There was no difference in the pGFs used when the object was moved to a lower platform compared with one of the same height (*p* = .34). No interaction was observed between object size and movement goal (*p* = .82).

With the pLF data, we observed a main effect of object size, *F*(1, 31) = 21.16, *p* < .001, ω^2^ = 0.041, suggesting that participants lifted the large objects with more force than they did the small objects (average mean difference of 0.16 N). We also observed a main effect of end goal, *F*(2, 62) = 12.63, *p* < .001, ω^2^ = 0.03. Post hoc comparisons (see Fig. 4b) showed that participants used more force to move the object to a higher platform than they did to a lower platform (*p* = .01, Cohen’s *d* = 0.55) or to a platform of the same height (*p* < .001, Cohen’s *d* = 0.79). There was no difference in the pLFs used when the object was moved to a lower platform compared with one of the same height (*p* = .20). No interaction was observed between object size and end goal (*p* = .18).

### Peak force rates used to lift objects

**Fig. 5:**
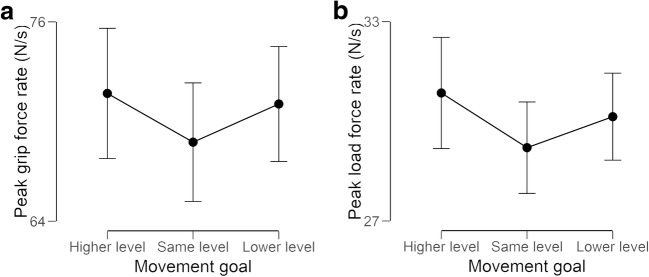
The (**a**) peak grip force rates and (**b**) peak load force rates used to move objects towards platforms of different heights, averaged across the small and large objects. Error bars show standard error of the mean, pooled across repeated-measures factors

With the pGFR data, we observed a main effect of object size, *F*(1, 31) = 44.23, *p* < .001, ω^2^ = 0.076, suggesting that participants gripped the large objects at a higher rate of force than the small objects (average mean difference of 9.8 N/s). In contrast to the forces themselves, there was no main effect of end goal (*p* = .059). As the *p* value for this main effect approached significance, we followed this test up with the same post hoc analyses as for the forces (see Fig. 5a), finding that a significantly higher rate of grip force was used to move the object to the higher level than to the same level (*p* = .023, Cohen’s *d* = 0.51), but not to the lower level (*p* > .99). There was no difference between the force rates used to move to the lower or to the same-level platform (*p* = .30), and no interaction was observed between object size and end goal (*p* = .60).

With the pLFR data, we observed a main effect of object size, *F*(1, 31) = 16.23, *p* < .001, ω^2^ = 0.027, suggesting that participants lifted the large objects at a higher rate of force than they did the small objects (average mean difference of 2.3 N/s). Here, in contrast to pGFR, we also observed a main effect of end goal, *F*(2, 62) = 3.47, *p* = .037, ω^2^ = 0.007. Post hoc comparisons (see Fig. 5b) showed that participants used a higher rate of force to move the object to a higher platform than to a platform of the same height (*p* = .022, Cohen’s *d* = 0.51), but not to the lower platform (*p* = .94). There was no difference in the pLFRs used when the object was moved to a lower platform compared with one of the same height (*p* = .39). No interaction was observed between object size and end goal (*p* = .19).

## Discussion

In this study, we examined whether moving an object to a higher platform, to a platform of the same height, or to a lower platform would affect how heavy it felt. Participants reported that moving objects to a higher platform made these objects feel, on average, heavier than when they were moved to either a lower platform or to a platform of the same height. This novel perceptual effect highlights the subjectivity of how humans experience the properties of objects in their environments.

Our experiment was carried out, in the context of the SWI, to provide insight into whether the perceptual effects of moving an object to a different-height platform might have the same cognitive underpinnings of prior expectations that drive weight illusions. In the context of the SWI, there are two clear observations we can make about this new perceptual effect. First, the increase in experienced weight that results from moving an object to a higher platform is notably smaller than the SWI. Second, the magnitude of the SWI was unaffected by the goal of the movement itself. Whereas at first glance this lack of interaction between our effects might suggest that the SWI is underpinned by a distinct mechanism from whatever drives the effect of an object feeling heavier when it is moved to a higher shelf, this is not necessarily the case. For example, given that the experience of a range of perceptual properties tends to unfold dynamically as more information is provided to the observer (Hick, 1952), it is quite plausible that these illusory changes in the experience of object weight may represent the same processes working sequentially. Follow-up work examining the temporal evolution of both heaviness perception with tight control of visual presentation of information, and alterations of instructions midmovement, may shed light on the shared underpinnings of these effect.

Interestingly, and contrary to prior work (Buckingham, Byrne, Paciocco, van Eimeren, & Goodale, 2014), these findings suggest that participants may be unable to disentangle feelings of heaviness from hedonic experiences of effort. Furthermore, although participants consistently reported that the small cylinder felt heavier than the large cylinder, their experience of the SWI was unaffected by the end goal of the movement. Thus, although the goal of a movement can influence how heavy an object feels when it is lifted, we find no evidence to suggest that this effect is underpinned by the same mechanism as the perceptual SWI. Indeed, in some sense this finding is in the opposite direction to the majority of weight-illusion work, where the hedonic experience contrasts prior expectations. Here, assuming that participants expect that moving an object to a higher shelf would be harder work, their subsequent experience of object heaviness seems to integrate with the sensory input of sensed mass. Similar integrative effects in the context of weight perception have been found in studies showing that a book or USB storage device feels heavier when the participant is made to believe it contains important information (Schneider, Parzuchowski, Wojciszke, Schwarz, & Koole, 2015; Schneider, Rutjens, Jostmann, & Lakens, 2011), suggesting that less direct cues to task effort (such as movement goals) might be integrated with sensory input in a distinct fashion from low-level cues to weight. Of course, in tasks such as this where little else is manipulated, it is possible that some findings may be driven by experimenter-expectancy effects, where participants are simply reporting what they feel the experimenter wishes them to report. Because participants are given no other instructions from the experimenter than the goal platform, it is also plausible that participants are simply reporting a different perceptual parameter than the one they claim to be experiencing—a notion frequently posited to explain the SWI (Ross & Di Lollo, 1970) . The perceptual findings reported herein would be markedly strengthened by conceptual replication in the context of other manipulations (e.g., physical mass, material) concurrently with task goal, and a more parametric manipulation of the task goal itself.

In addition to our perceptual findings, we noted similar trends in all our fingertip-force metrics. Participants gripped and lifted with more force when moving to the higher platform than to the same-height platform, and also applied these forces at a higher rate. At first glance, this might seem a trivial consequence of the task kinematics. However, in all conditions the initial movement made by participants was a vertical lift, so the move to the lower table surface in the lower platform condition contained no lateral slide. It is possible that these fingertip-force effects might reflect end state comfort effects, analogous to those described by Rosenbaum et al. (2012). Future work should aim to verify whether this paradigm (which has strong ecological validity in the context of object interaction) is related to more conventional end-state comfort tasks, and the degree to which the effects demonstrated in this study are binary (i.e., moving upwards elicits an automatic step increase in force and perception) or graded such that increasing apparent difficulty yields further gradual increases in forces and perceptions of heaviness and load forces. It is worth noting, of course, that none of the of the later kinematic differences associated with movements to higher or lower platforms would be captured by our dependent variables, which are largely pre-lift-off or capture only the earliest milliseconds of a lift. Although the peak force rates themselves are particularly robust in this regard, as they occur for the most part well before object lift-off (Johansson & Flanagan, 2009), we cannot rule out that the complex kinematic differences between our different conditions might have impacted the forces use to lift the objects in a preparatory fashion, and indeed the experience of object heaviness itself.

It would be parsimonious to suggest that the higher fingertip forces participants tended to use to lift to a higher platform might explain the perceptual illusion that objects feel heavier when they are being moved to a higher location. Indeed, this perceptual effect seems analogous to the one highlighted by Flanagan et al. (1995), where slippery objects feel heavier because of the extra effort expended in squeezing is harder to overcome the low-friction coefficient of an object covered in gel. Whether the tendency to apply more force when moving on object to a higher shelf is responsible for the perceptual effect is unclear, but the data do not strongly suggest this to be the case. Notably, the perceptual effect of the object feeling heavier when moved to a higher shelf is clear and robust in comparison with both the same and the lower platform, whereas the fingertip-force effects are only seen in comparison with the same-height platform. Of course, it is quite possible that the perceptual effects experienced by participants in this study, which are reported only after the movement is complete, are affected by distinct kinematics and energy expenditure profile variations of the upward and downward movements themselves in each condition. Future work manipulating the time of the perceptual report, or dynamically altering the goal of the movement during the lift itself, would be needed to fully disentangle the contributions of kinematics from fingertip-force application and prior expectations on perceptions of heaviness.

In summary, the current work aimed to examine whether making a more effortful-seeming movement (i.e., moving an object to a higher shelf) might influence a lifter’s experience of object weight. Contrary to what might be predicted based on typical weight-illusion paradigms, we found that moving an object to a higher shelf made it feel heavier than did moving it to a lower shelf or to a shelf of the same height. Future work should aim to determine the degree to which this novel perceptual effect stems from the cognitive anticipation of a potentially more difficult movement, the fingertip forces used to lift the objects themselves, or the distinct kinematics of the upwards, lateral, and downwards movements.
